# A Sensor Based Waste Rock Detection Method in Copper Mining Under Low Light Environment

**DOI:** 10.3390/s25195961

**Published:** 2025-09-25

**Authors:** Jianing Ding, Fuming Qu, Weihua Zhou, Jiajun Xu, Lingyu Zhao, Yaming Ji

**Affiliations:** 1 Institute of Minerals Research, University of Science and Technology Beijing, Beijing 100083, China; m202320067@xs.ustb.edu.cn (J.D.); m202420145@xs.ustb.edu.cn (W.Z.); m202510094@xs.ustb.edu.cn (J.X.); lingyulanazhao@xs.ustb.edu.cn (L.Z.); m202320089@xs.ustb.edu.cn (Y.J.); 2 School of Resources and Safety Engineering, University of Science and Technology Beijing, Beijing 100083, China; 3 School of Economics and Management, University of Science and Technology Beijing, Beijing 100083, China

**Keywords:** mineral processing, vision sensor, deep learning, object detection, low light

## Abstract

During production, copper mining could generate substantial waste rock that impacts land use and the environment. Advances in deep learning have enabled efficient, cost-effective intelligent sorting, where vision sensor performance critically determines sorting accuracy and efficiency. However, the sorting environment of copper mine waste rock is inherently complex, particularly within the conveyor belt section of the sorting machine, where insufficient and uneven lighting significantly impairs the performance of vision-based detection systems. To address the above challenges, a deep-learning-based copper mine waste rock detection algorithm under low-light environments is proposed. Firstly, an Illumination Adaptive Transformer (IAT) module is added as a preprocessing layer at the beginning of the Backbone to enhance the brightness of the images acquired by the vision sensor. Secondly, a Local Enhancement-Global Modulation (LEGM) module is integrated after the A2C2f and C3k2 modules in the Neck to enhance the detection accuracy. Finally, to further improve the model performance, MPDIoU is introduced to optimize the original loss function CIoU. As a result, the proposed algorithm achieved an mAP@0.5 of 0.957 and an mAP@0.5:0.95 of 0.689, outperforming advanced methods by 1.9% and 8.6%, respectively.

## 1. Introduction

Copper plays a crucial role in supporting the development of modern society, technology and infrastructure [1]. It is widely used in various industries, such as electric power, machinery, construction, transportation, etc. [2], making the efficient and sustainable utilization of copper ore resources particularly important. During the production process, waste rock should be separated from copper minerals as early as possible to conserve energy and time in subsequent processing steps. Therefore, sorting of copper ore waste rock is not only an urgent need for environmental protection but also an important strategy to achieve sustainable development of the mining industry [3,4,5].

With the rapid development of the global technological revolution and the advancement of industrialization, technologies in waste rock sorting are also evolving continuously. The traditional manual sorting model, characterized by high labor intensity, low efficiency, and poor precision under harsh working conditions, can no longer meet the demands of high-quality industry development. With the widespread use of deep learning in object detection, computer-vision-based intelligent sorting technology has emerged, providing an efficient solution for the automated sorting of copper mine waste rock [6,7]. However, practical implementation still faces significant technical challenges, the most significant being low and uneven lighting inside sorting machines. Such conditions make it difficult for image sensors to capture ore features accurately, leading to lower image quality, loss of detail, and frequent detection errors. Therefore, developing a real-time detection method for waste rocks on conveyor belts under low-light environments is critical for mineral processing development [8,9].

Current research on mineral waste rock sorting is primarily based on computer vision classification algorithms. With various image-based learning methods [10], it can preprocess input mineral images and construct classification models to achieve mineral sorting. For example, Liu Y et al. [11] designed a lightweight ore sorting network (LOSN), which integrates lightweight structures, attention mechanisms, and multi-scale feature fusion techniques to achieve end-to-end localization and classification of ore particles. Shatwell et al. [12] developed an algorithm based on image processing and machine learning, specifically designed for classifying high-grade ore rocks and low-grade waste rocks in gold and silver mines, enabling real-time ore sorting. Tsangaratos et al. [13] proposed a real-time mineral classification system that integrates classical computer vision techniques with advanced deep learning algorithms. This system can identify multiple minerals within a single frame and output probabilities for multiple mineral types, facilitating real-time mineral classification. However, existing methods heavily rely on the quality of input image. This dependence not only makes image acquisition challenging under harsh working conditions but also significantly increases recognition time and reduces sorting efficiency. Low-light or uneven-lighting conditions in actual sorting environments further degrade image quality, which makes feature extraction more difficult. In recent years, numerous scholars from various fields have developed methods to enhance real-time target detection in low-light environments by improving the input image quality, focusing primarily on image enhancement, small-target detection, noise suppression, and target deblurring.

Regarding image enhancement, Hui Y et al. [14] proposed the WSA-YOLO algorithm, a weakly supervised adaptive object detection algorithm for low-light environments based on YOLOv7. This algorithm decomposes images into reflectance and illumination maps for separate enhancement through a decomposition network, addressing performance degradation in object detection caused by the loss of fundamental image features under low-light conditions and the imbalance in low-level semantic information between low-light and normal images. Zhou Y et al. [15] introduced an improved spatial object detection method based on YOLO11, which enhances image quality by applying bilateral filtering to remove noise while preserving edge texture details and using Contrast Limited Adaptive Histogram Equalization (CLAHE) to improve image contrast. Zhang Q et al. [16] proposed the multi-module network LLD-YOLO for robust vehicle detection in low-light conditions. It enhances low-light images by integrating a DarkNet module based on self-calibrated illumination learning for adaptive illumination adjustment.

In the small target detection field, Zhang M et al. [17] proposed a Gaussian Curvature-Inspired Network (GCI-Net) for single-frame infrared small target detection, addressing the difficulty of separating small infrared targets from backgrounds with high brightness and strong edges in complex environments where they share similar physical characteristics. Wang S et al. [18] introduced a lightweight infrared small target detection algorithm called PHSI-RTDETR for drone aerial imagery, tackling issues of low model accuracy and high computational complexity caused by complex ground environments and uneven target scales in infrared image target detection. Wang Z et al. [19] proposed the PC-YOLO11s model to overcome detection difficulties in small target scenarios arising from factors such as small size, low resolution, weak contrast, and significant background interference. Fang, W. et al. [20] proposed a lightweight TBF-YOLOv8n tea bud detection model based on an improved YOLOv8n to address issues such as the high computational complexity of deep learning detection models and the difficulty of detecting tea buds due to their small size, complex background, and high density.

Regarding noise suppression and target blurring issues. Peng D et al. [21] proposed NLE-YOLO, a novel low-light object detection method based on YOLOv5 with fused feature enhancement, to overcome accuracy issues caused by insufficient illumination and noise interference in low-light environments. Das P P et al. [22] introduced YOLO-D, a domain-adaptive low-light object detection framework based on YOLOv8, designed to mitigate performance degradation in conventional detection systems due to low visibility, poor contrast, and high noise in low-light conditions. Liu Q et al. [23] developed an efficient object detection network architecture suitable for complex low-light environments, tackling low accuracy issues caused by blurring and low contrast during target detection under weak light. Han Z et al. [24] proposed 3L-YOLO, a lightweight low-light object detection algorithm based on YOLOv8n, addressing performance degradation and high computational resource consumption resulting from low contrast, high noise, and blurred boundaries in low-light detection. Ye S et al. [25] presented YES, a novel low-light object detection framework, to resolve the “target-background confusion” issue caused by uneven illumination in dim environments. Qi Z et al. [26] proposed the HSG-Net model, based on the lightweight YOLOv8, aiming to enhance detection accuracy and reduce computational costs in variable lighting conditions and complex backgrounds.

Additionally, some researchers have focused on improving real-time performance and computational efficiency from a methodological perspective rather than relying solely on input quality. Zhao Y et al. [27] proposed a Real-time Efficient Enhancement Network (RTEE-Net) for low-light images, addressing the trade-off between model complexity and performance in low-light image enhancement. Qiu Z et al. [28] introduced GS-YOLO-Seg, an improved YOLO11-Seg-based method, to resolve issues of low efficiency in traditional approaches and performance bottlenecks on edge devices during low-grade graphite ore sorting. Li J et al. [29] developed an enhanced model based on YOLOv5, featuring a parallel convolutional architecture, which improves the accuracy and efficiency of photovoltaic panel detection by addressing challenges arising from low resolution, small target sizes, and complex backgrounds in remote sensing imagery.

Based on a comprehensive analysis of existing research, various algorithms demonstrate certain advantages within their respective low-light application contexts. However, some inadequately extract comprehensive features when sorting copper minerals in complex and dim environments. Others are tailored to particular scenarios and struggle to adapt to specialized operational conditions such as conveyor belts, while certain methods impose demanding hardware requirements due to high model complexity, failing to meet real-time sorting needs.

This study proposes a novel method for identifying waste rock in copper ore within conveyor systems, specifically designed for operation under low-light conditions. Industrial experiments were conducted in environments free from overexposure or significant noise interference. Furthermore, the use of high-frame-rate industrial cameras minimized image distortion caused by abnormal exposure and noise. Consequently, the core objective of this research is to overcome recognition challenges in low-light environments. To address issues such as low detection accuracy, high false negative rates, and high false positive rates caused by blurred images resulting from dim lighting and uneven illumination, we introduce the YOLO-ILM algorithm. This algorithm enables real-time detection within low-light conveyor belt environments. Building upon the advanced YOLOv12 framework [30], YOLO-ILM leverages its efficient feature extraction and target localization capabilities. The algorithm further integrates a series of image enhancement and feature optimization techniques specifically designed for low-light and blurry scenarios. Through synergistic module collaboration, it significantly enhances adaptability to complex lighting conditions while ensuring detection speed meets the industrial requirements. The model introduces the following innovations:

(1) To address image quality degradation caused by low-light conditions, an Illumination-Adaptive Transformer module is proposed and integrated as a preprocessing layer at the network front-end to enhance the brightness of subsequent input images.
(2) To improve the detection accuracy of copper ore waste rocks, a method is introduced that integrates local feature embedding into global feature extraction modules following the A2C2f and C3k2 modules.
(3) To further enhance the detection accuracy of copper ore waste rocks, the original loss function has been refined, optimizing the network.

## 2. Materials and Methods

**Figure 1:**
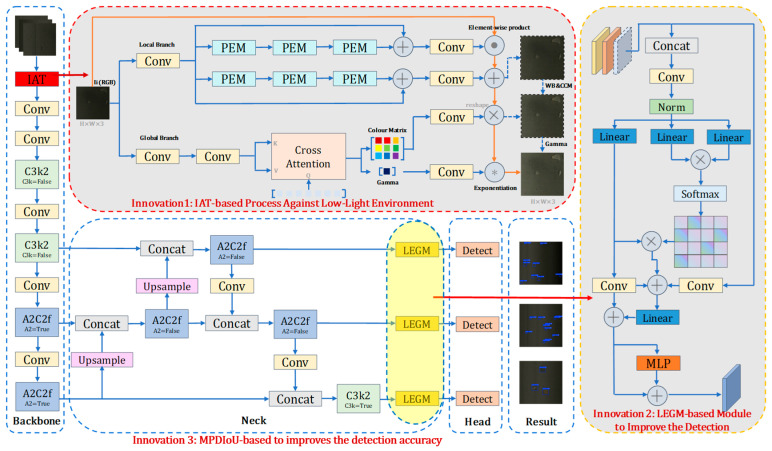
YOLO-ILM Model Architecture Diagram (The red arrow represents the newly added module, whereas the remaining arrows indicate connections between the existing workflow steps.).

The proposed YOLO-ILM algorithm for low-light copper mine waste rock detection is depicted in Figure 1, which consists of three core components: the Backbone, the Neck, and the Head.

In the Backbone section, to address the issue of poor input quality under low-light conditions, the Illumination Adaptive Transformer module (IAT) [31] is incorporated as a preprocessing layer at the front-end of the Backbone. This module effectively mitigates the adverse effects of uneven illumination, thereby providing high-quality input for subsequent copper mine waste rock detection tasks. Its lightweight design also ensures efficient operation under computational resource constraints.

In the Neck section, the preprocessed waste rock images from copper mines are input into the Backbone for feature extraction after IAT preprocessing. The extracted features subsequently enter the Neck for feature fusion. To more comprehensively integrate multi-scale features and enhance the model’s discriminative capability for waste rock characteristics, the Local Feature-Embedded Global Feature Extraction Module (LEGM) [32] is integrated after both the A2C2f and C3k2 modules within the Neck. By combining self-attention and convolution, it integrates local and global features; by aggregating multi-source inputs and depth information, it achieves multi-scale feature fusion. This enables the model to adaptively learn dependencies between different features, enhances focus on critical regions, and suppresses noise interference. The fused features are then forwarded to the Head for final prediction.

In the Head section, the Minimum Points Distance IoU (MPDIoU) loss function [33] is introduced, which places greater emphasis on the positional relationships between bounding boxes and their overlapping regions. This further improves the detection accuracy for copper mine waste rock.

### 2.1. Illumination Adaptive Transformer Module

Copper mine waste rock detection in real-world environments suffers from low-light and insufficient-light conditions, resulting in degraded visual quality that adversely affects downstream computer vision tasks. Images captured under weak or insufficient lighting exhibit darkness due to limited photon counts and in-camera noise. Therefore, it is necessary to pre-integrate an illumination-adaptive preprocessing module. The method of “optimizing input quality first and then improving model performance” has been verified in many fields [34]. Numerous solutions have been proposed in existing research, such as low-light enhancement or methods improving robustness in low-light and over-exposed conditions with oversaturation. However, these approaches have limitations and shortcomings. Low-light enhancement methods, while recovering details and suppressing accompanying noise, may inadvertently obscure fine details. Furthermore, when local image areas are severely over-exposed (pure white) or under-exposed (pure black), the original scene’s brightness information is irretrievably lost. Methods enhancing robustness to oversaturation under low-light and over-exposure conditions suffer from high parameter complexity and significant computational cost.

**Figure 2:**
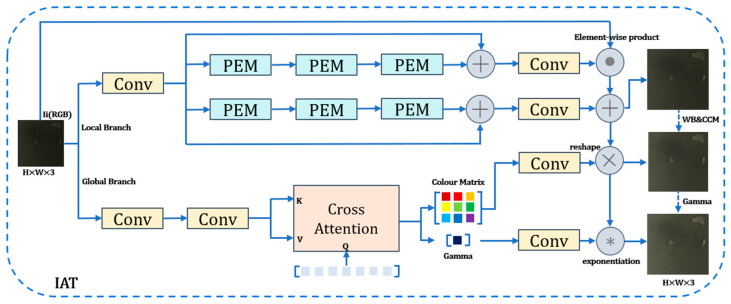
IAT Structure Diagram.

In contrast, the Illumination Adaptive Transformer (IAT) addresses both low-light enhancement and exposure correction through its designed lightweight dual-branch structure. IAT simulates the camera Image Signal Processor (ISP) pipeline, as illustrated in Figure 2, dividing image enhancement into two complementary components: local pixel-level illumination adjustment and global dynamic adjustment of ISP parameters.

For an input sRGB image Ii captured under lighting condition Li, illumination-induced degradation is addressed through the Illumination Adaptive Transformer (IAT) module via its two core components: local pixel-level adjustment and global ISP parameter adjustment.

First, illumination effects are corrected while preserving image details via the Local Branch. This branch employs a transformer-style architecture, processing the image while maintaining the input resolution. Its core consists of stacked Pixel Enhancement Modules (PEMs). Each PEM first encodes positional information using a 3 × 3 depth-wise convolution. Subsequently, a Pointwise Convolution (PWConv)—Depth-wise Convolution (DWConv)—Pointwise Convolution (PWConv) structure enhances local details. Two separate 1 × 1 convolutions then strengthen feature representations. Innovatively, standard Layer Normalization is replaced with Illumination Normalization (I Norm), which learns scaling factor a and bias term b applied before channel fusion. Three PEM modules are stacked within the branch. Their outputs are fused with the input features via skip connections (element-wise addition), effectively preserving original details. Finally, a 3 × 3 convolution reduces dimensionality, followed by the application of ReLU (yielding a multiplicative map M) and Tanh (yielding an additive map A) activation functions. This design achieves a balance between lightweight implementation and detail enhancement.

Second, parameter modulation is performed by the Global Branch. This branch forms the core of the IAT model for handling ISP pipeline operations and generating the target sRGB image. It focuses on capturing global interactions while maintaining lightweight efficiency. The process is as follows: First, a lightweight encoder, composed of two stacked convolutional layers, encodes the input image. This design reduces feature map resolution to minimize computational cost while extracting high-dimensional global features. The encoded features are then fed into the Global Prediction Module (GPM). Within the GPM, a globally initialized component query (Q) attends to keys (K) and values (V) generated from the encoded features. Notably, positional encoding for K and V is implemented via depth-wise convolution, enabling flexible adaptation to varying input resolutions. The features are subsequently processed by a Feed-Forward Network (FFN) containing two linear layers. Finally, two specially initialized parameters are appended, outputting the joint color transformation matrix (W) and the gamma correction parameter (γ). The initialization strategy (setting the color matrix to the identity matrix and γ to 1) effectively ensures training stability. This entire structure dynamically controls global ISP-related parameters (color correction, gamma correction) through a transformer-style query mechanism. This enables adaptation to diverse lighting conditions while rigorously maintaining the model’s lightweight nature and computational efficiency.

When the IAT module is not incorporated into the model, the image input is expressed as shown in Equation (1):

(1) $$Input=I_{i}\;=I_{raw}$$

After incorporating the IAT module into the model, the preprocessed image output by the IAT module is expressed as shown in Equation (2):

(2) $$I_{t}\;=max{\left(\sum_{cj}W_{ci,cj}\;(I_{i}\;⊙M+A),0\right)}^{\gamma }$$

**Figure 3:**
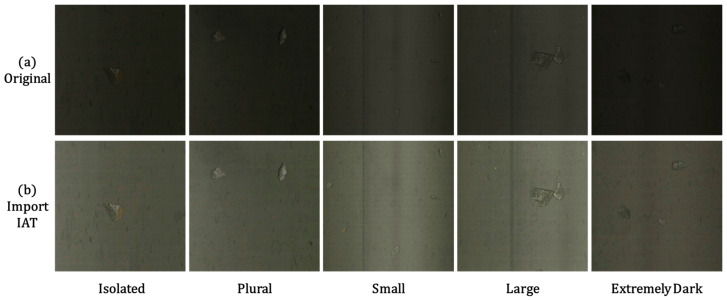
Effect Diagram Before and After IAT Processing.

Functioning as a preprocessing layer, the IAT module transforms the raw input *I_raw_* into an illumination-enhanced image, facilitating subsequent feature extraction by convolutional layers. The enhancement effect is visualized in Figure 3. Figure 3a,b depicts the original image and the IAT-processed result (denoted as IAT Output), respectively. Across diverse scenarios—including Isolated (single waste rock), Plural (multiple waste rocks), Small (smaller waste rocks), Large (larger waste rocks), and notably Extremely Dark (severe low-light conditions commonly encountered in areas like conveyor belt edges where illumination is insufficient and ambient brightness is critically low)—the comparison between original and processed images demonstrates the IAT module’s pronounced enhancement effect on low-illumination imagery. Additionally, this study employs Peak Signal-to-Noise Ratio (PSNR) and Structural Similarity Index (SSIM) as primary metrics for evaluating image enhancement performance. Higher PSNR values indicate smaller differences between the enhanced image and the reference image, while SSIM values closer to 1 signify stronger consistency in image structure and texture. Table 1 presents experimental comparisons between IAT and representative enhancement methods on this dataset. These results further validate IAT’s superior image enhancement performance in this study.

### 2.2. LEGM Attention Mechanism

Building upon the base model, the LEGM attention mechanism is introduced to enhance both detail preservation and global context modeling. The fundamental principle of self-attention mechanisms is that when processing sequential data, each element can establish relationships with any other element in the sequence, rather than relying solely on adjacent elements. It adaptively captures long-range dependencies by calculating the relative importance between elements. Consequently, the self-attention module computes association weights between all positions in the feature map, dynamically aggregating global information.

However, conventional self-attention suffers from a key limitation: it focuses exclusively on global dependencies while neglecting local details, often resulting in structurally blurred restored images. To address this, the LEGM module integrates convolutional layers for local feature embedding. This design combines the strengths of convolutional layers—excellent at local feature extraction—with the self-attention module’s ability to model global context. By fusing these capabilities, LEGM effectively models global haze distribution patterns while preserving fine details, thereby significantly enhancing the visual realism of the dehazed results.

**Figure 4:**
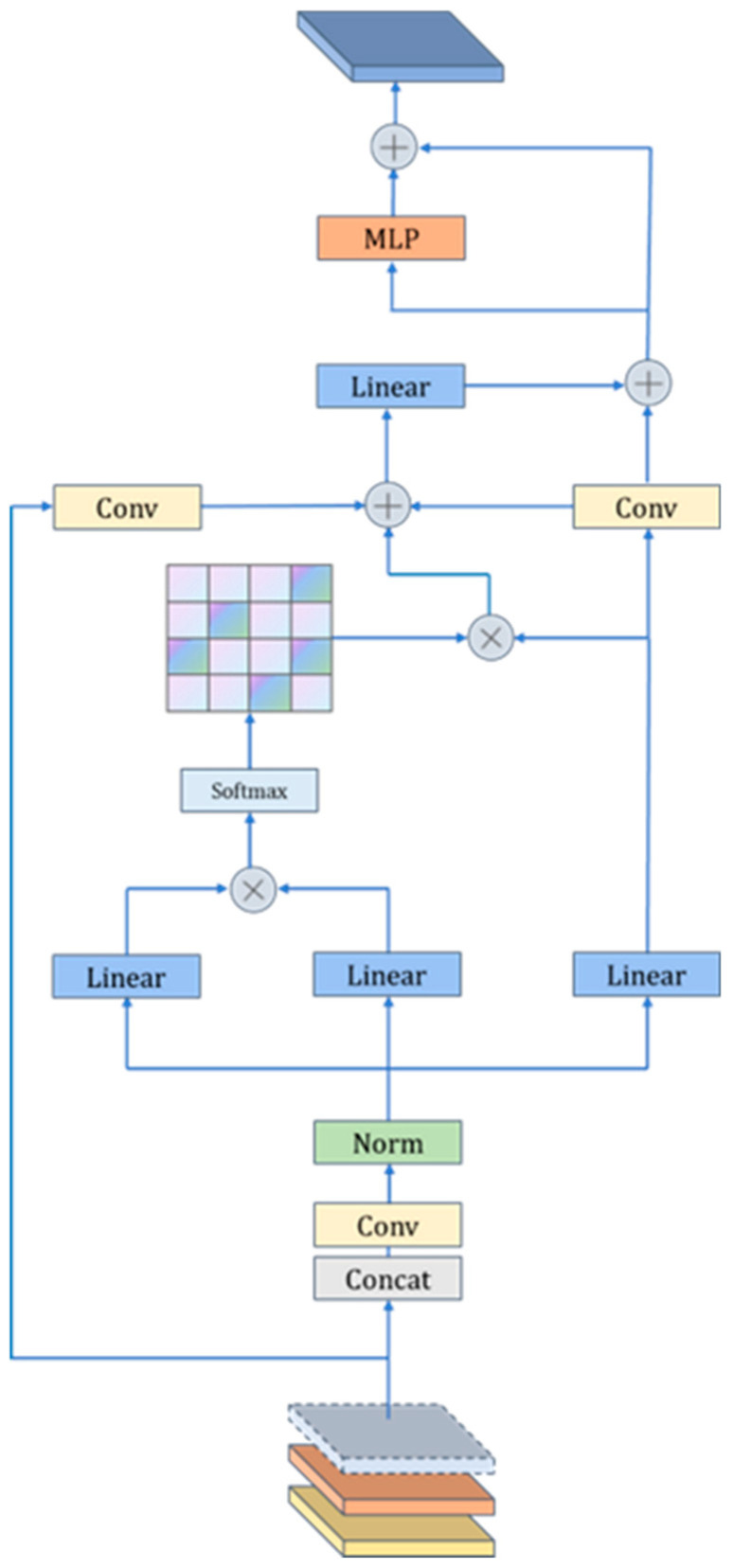
LEGM Structure Diagram.

The LEGM module integrates a self-attention mechanism with a convolutional module. It concurrently receives and concatenates shallow, mid-level, and deep features. During the feature processing stage, the concatenated features first pass through convolutional layers and normalization layers to explicitly extract local structures, thereby overcoming the inherent local receptive field limitations of traditional self-attention. These features subsequently transform a Multilayer Perceptron (MLP), are further processed by a linear layer, and finally pass through a Softmax function to generate the attention weight matrix. In the feature fusion stage, this attention weight matrix is element-wise multiplied with the input features. The resulting features are then processed through convolutional and linear layers. To enhance the precision of attention focus—particularly on distant, high-haze-concentration areas—these processed features are additively combined with the original input features via a residual connection. Following this, a filtering operation is applied, and the features are finally output through another Multilayer Perceptron (MLP). By synergistically fusing convolution-based local feature embedding with depth-prior-guided self-attention, LEGM effectively achieves a balance between preserving local details and capturing global structural information for the image dehazing task, as illustrated in Figure 4.

Fusion of input features:

(3) $$F_{in}\;=Concat(Conv1\times 1(F_{unet}\;),Conv3\times 3(F_{unet}\;),DRDB(F_{depth}\;))$$

Self-attentive module:

(4) $$Q=Conv(F_{in}\;),K=Conv(F_{in}\;),V=Conv(F_{in}\;),d_{K}\;=dim(K)$$

(5) $$F_{att}=\mathrm{Softmax}\left(\frac{QK^{T}}{\sqrt{d_{K}}}\right)V$$

MLP transformation:

(6) $$F_{mlp}=MLP(F_{att})$$

Output Equation:

(7) $$F_{out}=Conv\left(LayerNorm\left(F_{in}+F_{mlp}\right)\right)$$

Following LEGM integration, the network output is:

(8) $$\left\{\begin{array}{l} F_{out1}=LEGM\left(A2C2f(F_{in1})\right)\\ F_{out2}=LEGM\left(A2C2f(F_{in2})\right)\\ F_{out3}=LEGM\left(C3K2(F_{in3})\right) \end{array}\right.$$

After incorporating the LEGM module into the original model, the self- attentive module integrates the locally extracted convolutional features with global contextual information, enhancing feature representation capabilities and improving the utilization efficiency of image structure and depth information.

### 2.3. MPDIoU Loss Function

The original CIoU [38] loss function in the model considers both center-point distance and aspect ratio. However, it focuses solely on relative aspect ratio deviation rather than absolute size differences, severely constraining regression accuracy and convergence efficiency when predicted and ground-truth bounding boxes share identical aspect ratios but differ significantly in scale. In contrast, the MPDIoU loss function currently employed in our model fundamentally resolves this limitation by innovatively minimizing the distances between corresponding top-left and bottom-right corner points of predicted and ground-truth bounding boxes. This approach unifies the geometric properties of bounding boxes (defined by four corner coordinates) into a concise metric that comprehensively incorporates critical factors, including overlap area, center-point distance, and width-height deviations, while directly optimizing absolute size differences. Simultaneously, it streamlines the computational process, delivering an efficient and robust solution for bounding box regression.

The final CIoU loss is computed as follows:

(9) $$L_{CIOU}=L_{base}+\alpha \times \frac{4}{\pi^{2}}{\left(\arctan\frac{w_{1}}{h_{1}}-\arctan\frac{w_{2}}{h_{2}}\right)}^{2}$$

where $L_{base}$ denotes the fundamental geometric error, α represents the dynamic weighting parameter, and their computational formulations are given, respectively, as follows:

(10) $$L_{base}=(1-IoU)+\frac{\rho^{2}(M_{2},M_{1})}{c^{2}}$$

(11) $$\alpha =\left\{\begin{array}{ll} 0, & \mathrm{if}\;IoU<0.5,\\ \frac{V}{(1-IoU)+V}, & \mathrm{if}\;IoU\geq 0.5. \end{array}\right.$$

where *M*_1_ = (*x^A^*, *y^A^*) and *M*_2_ = (*x^B^*, *y^B^*) denote the center coordinates of ground-truth box *A* and predicted box *B*, respectively; C represents the minimum enclosing rectangle covering both boxes, ρ corresponds to the Euclidean distance between *M*_1_ and *M*_2_. *w*_1_ and *w*_2_ indicate the widths of boxes A and B; *h*_1_ and *h*_2_ signify their corresponding heights.

The MPDIoU loss function is formulated as follows:

(12) $$L_{MPDIoU}=1-MPDIoU$$

The core computational formulation of MPDIoU is defined as follows:

(13) $$MPDIoU=IoU-\left(\frac{d_{1}^{2}}{w^{2}+h^{2}}+\frac{d_{2}^{2}}{w^{2}+h^{2}}\right)$$

where IoU measures the overlap, and *d*_1_^2^ and *d*_2_^2^ represent the distances from the top-left corner and the bottom-right corner, respectively. These are calculated as follows:

(14) $$IoU=\frac{I}{U}\;$$

(15) $$d_{1}^{2}={\left(x_{1}^{B}-x_{1}^{A}\right)}^{2}+{\left(y_{1}^{B}-y_{1}^{A}\right)}^{2}\;$$

(16) $$d_{2}^{2}={\left(x_{2}^{B}-x_{2}^{A}\right)}^{2}+{\left(y_{2}^{B}-y_{2}^{A}\right)}^{2}$$

**Figure 5:**
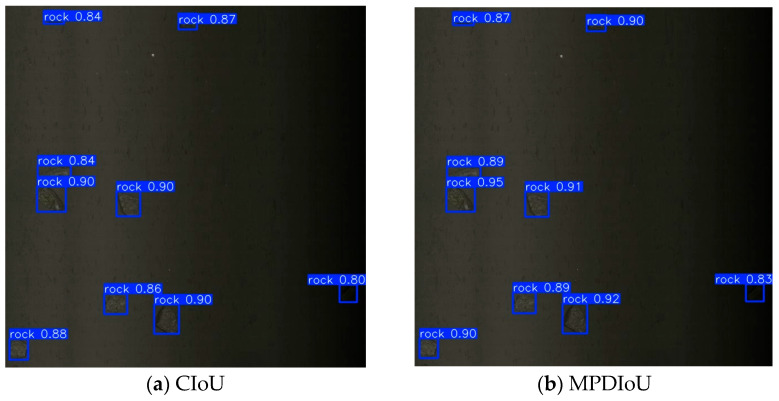
Prediction Box Comparisons.

Consequently, when employed in object detection, MPDIoU offers significant advantages. By comprehensively accounting for overlapping and non-overlapping regions, center point distance, and width-height deviations, it effectively resolves optimization inefficiencies that occur when bounding boxes share identical aspect ratios but differ in absolute dimensions, enabling precise discrimination between predicted boxes located inside and outside the ground truth. Furthermore, its computational efficiency stems from determining relevant factors directly from the four bounding box coordinates, minimizing computational overhead, and facilitating straightforward integration into detection algorithms. Finally, as shown in the Figure 5, MPDIoU accelerates training convergence, thus achieving higher accuracy in shorter timeframes while also delivering superior final detection precision.

### 2.4. Evaluation Metrics

To better evaluate the comprehensive performance of each algorithm, based on the specific characteristics of the copper ore waste rock detection scenario, this paper selects three primary evaluation metrics: Precision (P), Recall (R), and Mean Average Precision (mAP), where:

(1) Precision (P) refers to the proportion of true positive samples among all samples predicted as positive by the model. A higher precision indicates a lower probability of misjudgment by the model. Its calculation formula is:

(17) $$\mathrm{Precision}=\frac{\mathrm{TP}}{\mathrm{TP}+\mathrm{FP}}$$

In the formula, TP (True Positive) represents the number of samples correctly predicted as positive by the model, i.e., samples that are positive and predicted as positive; FP (False Positive) represents the number of samples incorrectly predicted as positive by the model, i.e., samples that are negative but predicted as positive.

(2) Recall (R) refers to the proportion of actual positive samples correctly predicted as positive by the model among all actual positive samples. A higher recall indicates a lower probability of missed detections by the model. Its calculation formula is:

(18) $$\mathrm{Recall}=\frac{\mathrm{TP}}{\mathrm{TP}+\mathrm{FN}}$$

In the formula, FN (False Negative) represents the number of samples incorrectly predicted as negative by the model, that is, samples that are positive but predicted as negative.

(3) Average Precision (AP) refers to the area under the Precision-Recall curve. A higher AP indicates better model precision, recall, and overall performance. Mean Average Precision (mAP) is the average of AP values across all categories. The calculation formulas for AP and mAP are, respectively, as follows:

(19) $$\mathrm{AP}=\int_{0}^{1}P(R)dR$$

(20) $$\mathrm{mAP}=\frac{1}{M}\sum_{i=1}^{M}{\mathrm{AP}}_{i}$$

In the formula, AP_i_ represents the average detection accuracy of the model for a specific category; M represents the total number of categories.

## 3. Results

### 3.1. Experimental Dataset

**Figure 6:**
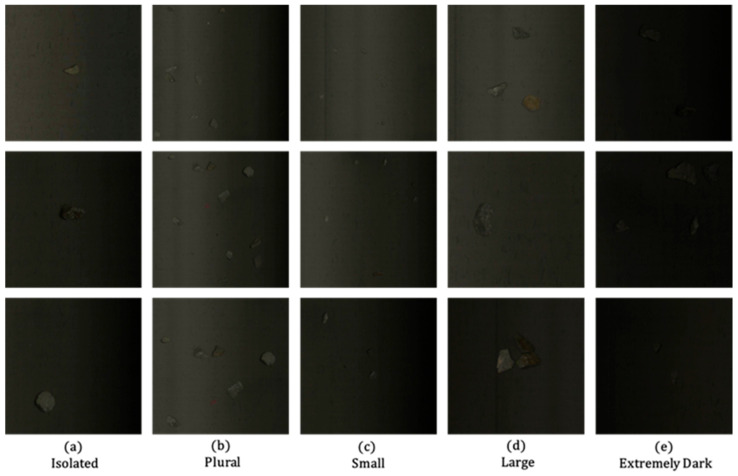
Instance Diagram.

The dataset used in this experiment is a copper ore waste rock dataset collected from the sorting site, which contains a total of 1200 images of copper ore waste rocks on the conveyor belt. To fully evaluate the performance of the algorithm and model, the dataset is divided into a training set, a validation set, and a test set in an 8:1:1 ratio. Specifically, the training set includes 960 images, the validation set contains 120 images, and the test set comprises 120 images. Some samples of the dataset are shown in Figure 6.

### 3.2. Experimental Environment and Parameters

In this experiment, four mainstream object detection algorithms were selected: Faster R-CNN [39], YOLOv8 [40], YOLOv11 [41], and YOLOv12. During the experiment, each algorithm was loaded onto the copper ore waste rock dataset for training, ultimately resulting in four distinct object detection models.

The hardware and software environment for this experiment are detailed in Table 2. In terms of hardware, the core computing device featured a central processing unit (CPU) of Intel Core Ultra 9-275HX (Intel Corporation, Santa Clara, CA, USA) and a graphics processing unit (GPU) of NVIDIA GeForce RTX 5080 (NVIDIA Corporation, Santa Clara, CA, USA) discrete graphics card, equipped with the CUDA platform and its corresponding cuDNN library. On the software side, the experiment was conducted on the Windows 11 operating system, utilizing the Python programming language and the PyTorch deep learning framework. PyCharm was chosen as the integrated development environment for writing, debugging, and executing the code.

To fully leverage the computational capabilities of the graphics processor used in the experiment and balance model training speed, this experiment sets the input image size to 640 × 640, the number of training epochs to 200, and the batch size to 32, ensuring efficient model training.

To optimize the training process, the stochastic gradient descent (SGD) algorithm was selected as the optimizer. To enhance the convergence speed and stability of SGD, the momentum mechanism was employed, with the momentum parameter set to 0.937. To prevent the computer from allocating excessive computational resources at once—which could lead to exceeding the upper limit of the graphics processor’s memory and cause Out of Memory (OOM) errors—the total number of GPU worker threads was set to 4. This configuration balances computational resource allocation and ensures stable model training.

### 3.3. Comparative Experiments

**Figure 7:**
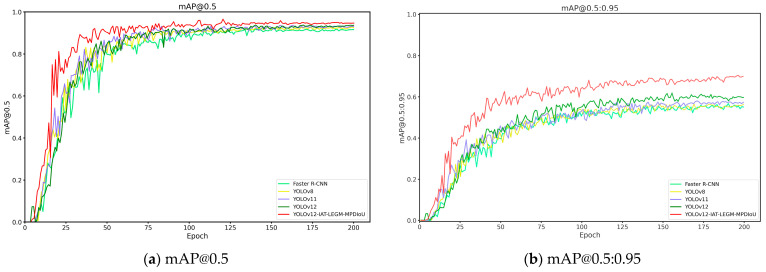
Experimental Plots under Different Models.

To fully validate the superiority of the YOLO-ILM model algorithm, the experiment selected Faster R-CNN, YOLOv8, YOLOv11, and YOLOv12 as comparative models. The experimental results of training and prediction on the copper ore waste rock dataset were used for comparison. The experimental outcomes from training different models are presented in Table 3 and Figure 7. Table 3 displays the Precision (P), Recall (R), and mAP evaluation metrics for each model, while Figure 7 illustrates the mAP@0.5 and mAP@0.5:0.95 results curves across the models.

Combining Table 3 and Figure 7, it is evident that among all evaluated models, the YOLO series models collectively outperform Faster R-CNN in precision, recall, and mean average precision (mAP). Specifically, YOLO-ILM achieved optimal performance across four key evaluation metrics: precision (*p* = 0.948), recall (R = 0.941), mAP@0.5 (0.957), and mAP@0.5:0.95(0.689). Compared to the original model, it improved mAP@0.5 and mAP@0.5:0.95 by 1.9% and 8.6%, respectively. Additionally, as evidenced by inference time, although the YOLO-ILM model is 2.7 ms slower than the baseline model, our model can meet real-time detection requirements.

**Figure 8:**
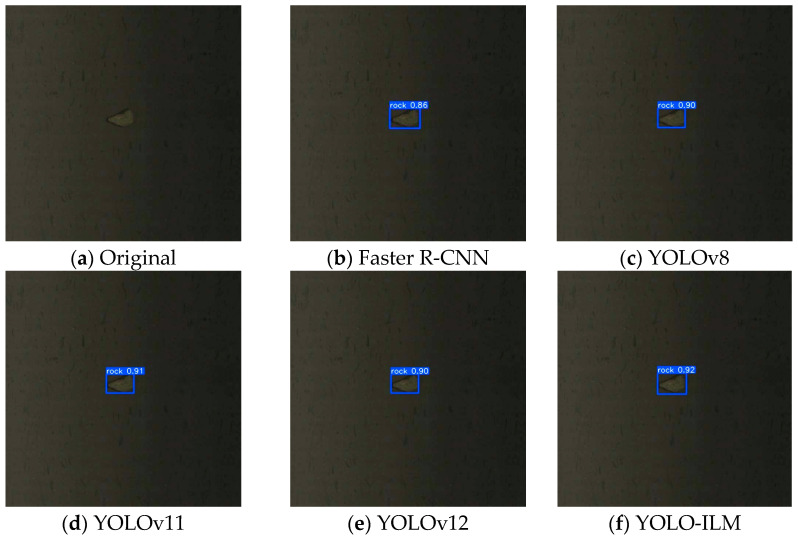
Isolated Detection Results.

**Figure 9:**
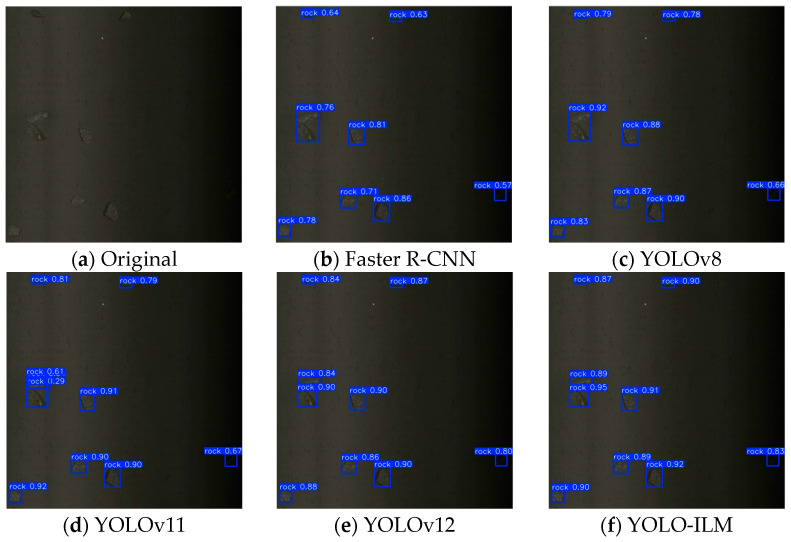
Plural Detection Results.

**Figure 10:**
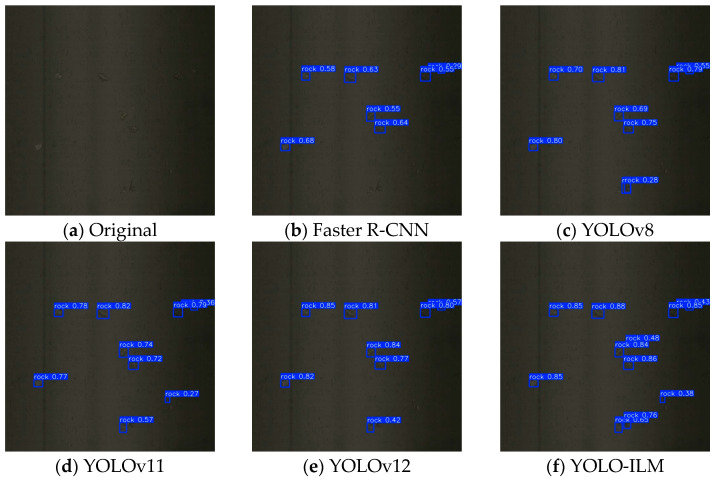
Small Detection Results.

**Figure 11:**
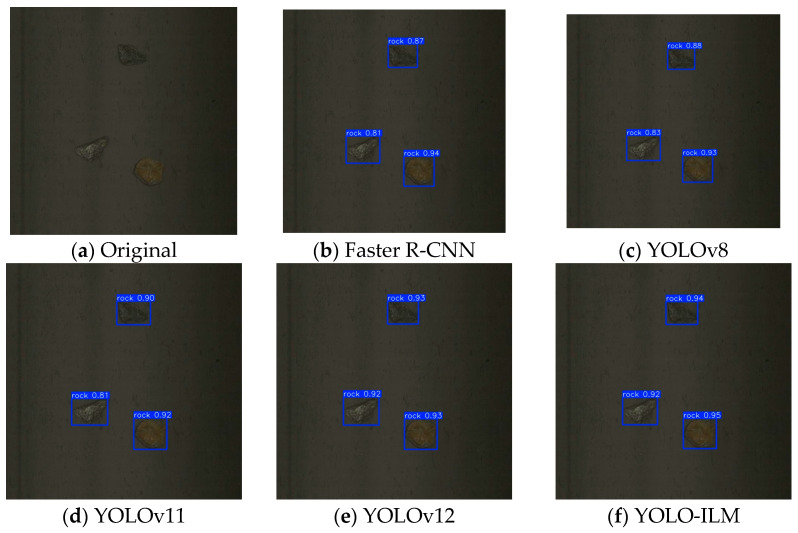
Large Detection Results.

**Figure 12:**
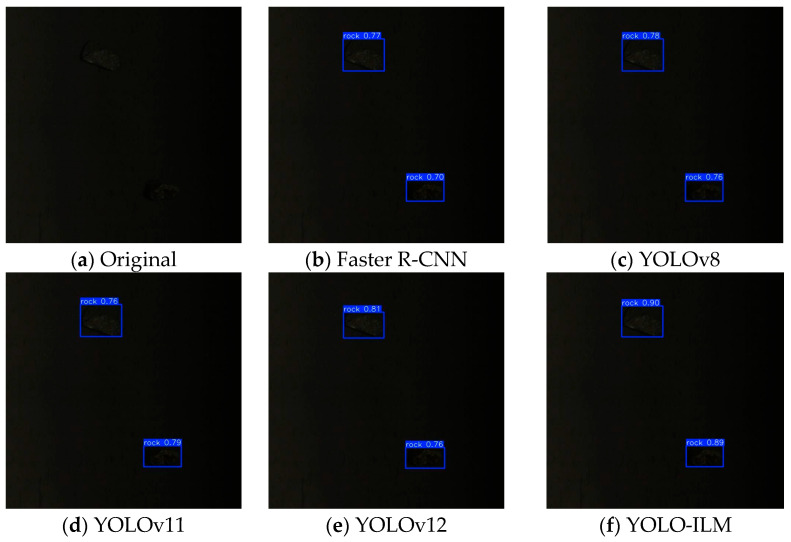
Extremely Dark Detection Results.

To further validate the effectiveness of the YOLO-ILM algorithm, the trained model is deployed to an on-site sorting machine and conducted field tests in complex low-light environments. The experimental results are shown in Figure 8, Figure 9, Figure 10, Figure 11 and Figure 12, where “rock” denotes detected copper ore waste. The results demonstrate that for multiple targets, Faster R-CNN and YOLOv8 exhibited partial missed detections (Figure 9) with low confidence, while YOLOv11 and YOLOv12 detected all targets, their confidence was lower than YOLO-ILM. Through comparison with other algorithm models, this fully demonstrates YOLO-ILM’s advanced performance in low-light target detection and its suitability for this task.

### 3.4. Ablation Experiments

**Figure 13:**
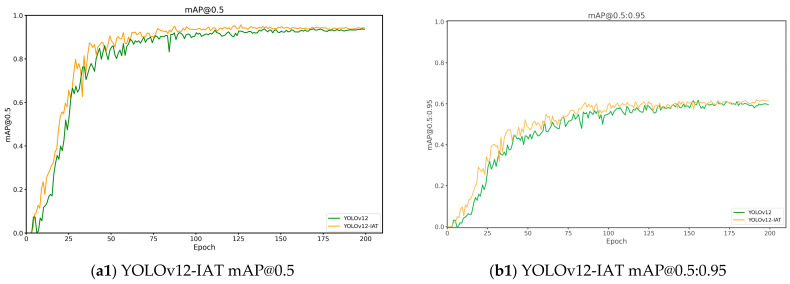

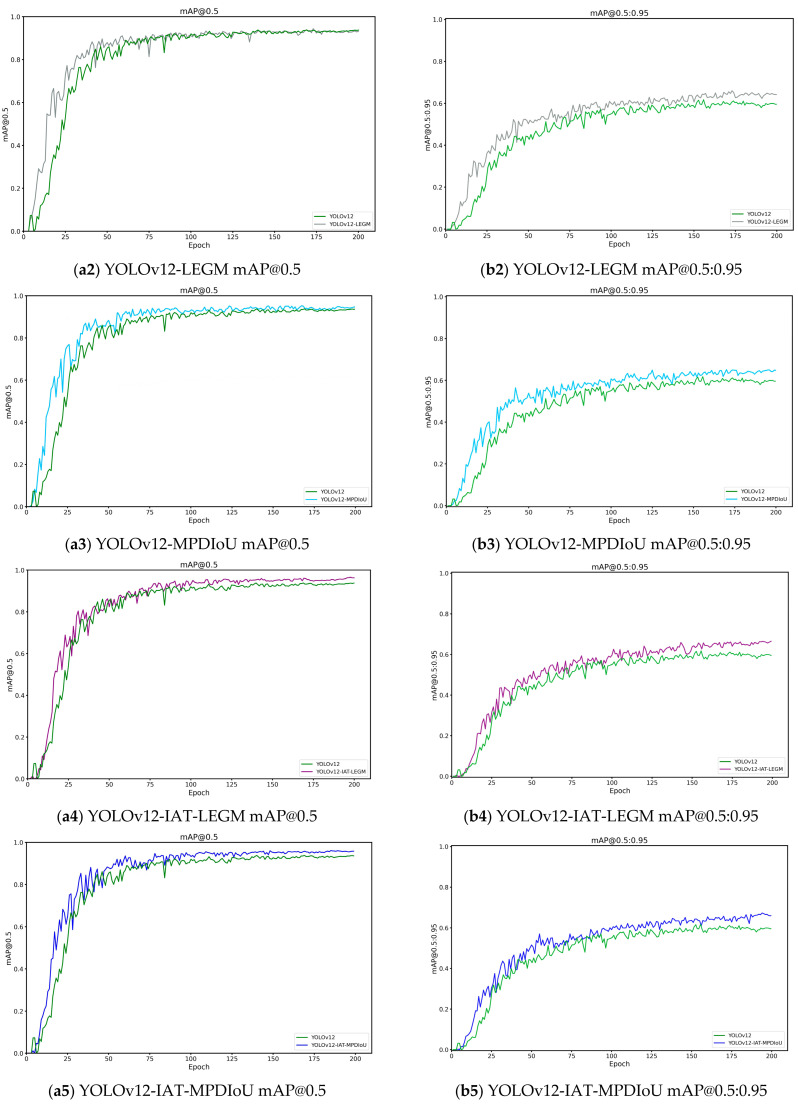

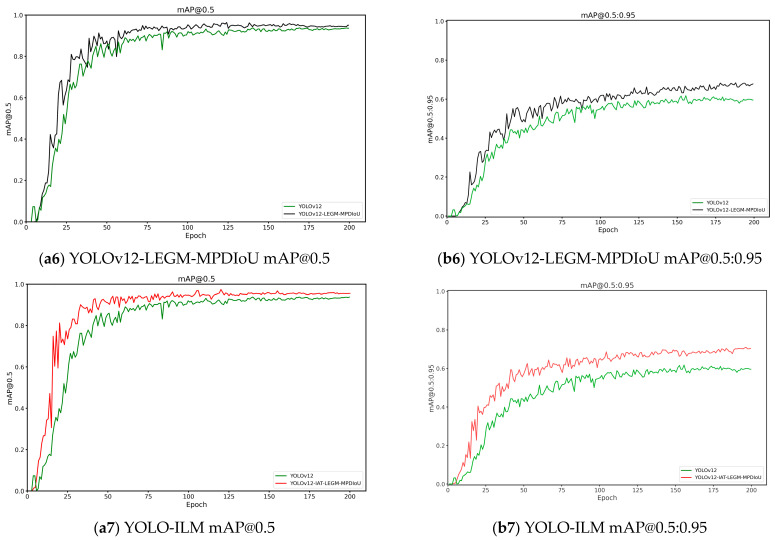
Performance Comparison of Ablation Experiments.

To further validate the effectiveness of each module (IAT, LEGM, MPDIoU) in the proposed algorithm, ablation experiments were conducted on the added modules. The experimental results are shown in Table 4 and Figure 13. In Table 4, using the YOLOv12 model as the baseline, the effectiveness of each improvement method was evaluated by incrementally adding one enhancement at a time. The symbol “√” indicates that the corresponding improvement module has been incorporated into the model. Figure 13 presents a comparative analysis of mAP@0.5 and mAP@0.5:0.95 results between various model combinations in the ablation experiments and the baseline model.

Combining Table 4 and Table 5, and Figure 13, initially, the baseline model YOLOv12 was tested, achieving mAP@0.5 of 0.938 and mAP@0.5:0.95 of 0.603. Separately introducing the IAT module, LEGM attention mechanism, and MPDIoU loss function each increased both mAP@0.5 and mAP@0.5:0.95 compared to the baseline model, indicating individual contributions from each module. Next, pairwise combinations of modules demonstrated further improvements in mAP@0.5 and mAP@0.5:0.95, with a greater magnitude than single modules alone. This demonstrates synergistic effects when modules are combined pairwise, significantly enhancing the model’s object detection performance. Finally, integrating all three improved modules achieved peak results: precision (*p* = 0.948), recall (R = 0.941), mAP@0.5(0.957), and mAP@0.5:0.95(0.689), representing improvements of 1.9% and 8.6% in mAP@0.5 and mAP@0.5:0.95, respectively, over the baseline model. These results confirm maximal synergistic gains when all three modules operate jointly, delivering comprehensive optimal performance across all evaluation metrics.

## 4. Conclusions

This study addresses the issue of low target detection accuracy caused by poor image quality in low-light environments during copper ore waste rock processing. YOLO-ILM, a real-time detection algorithm for low-light copper ore waste, is proposed with core contributions including:

(1) Integrating the YOLOv12 network with the Illumination Adaptive Transformer module IAT to enhance model adaptability to low-light conditions;
(2) Introducing the Locally Embedded Global-feature attention mechanism LEGM to improve target feature capture capability under complex environmental interference;
(3) Introducing an efficient and accurate bounding box regression loss function MPDIoU to reduce computational load while optimizing localization accuracy.

This method satisfies real-time detection requirements for copper ore waste in low-light scenarios. The experimental results demonstrate that through comparison with different detection algorithms, the proposed YOLO-ILM model shows significant advantages over Faster R-CNN, YOLOv8, YOLOv11, and YOLOv12 in both subjective visual detection effects and objective evaluation metrics. Compared to the baseline model, YOLO-ILM improves mAP@0.5 and mAP@0.5:0.95 by 1.9% and 8.6%, respectively, confirming the method’s superiority for real-time copper ore waste detection under complex conditions.

## Figures and Tables

**Table 1:** Quantitative Comparison of IAT and Other Mainstream Methods.

Method	Test 1		Test 2		Test 3		Test 4		Test 5	
	PSNR	SSIM	PSNR	SSIM	PSNR	SSIM	PSNR	SSIM	PSNR	SSIM
Zero-DCE [35]	9.207	0.515	9.429	0.528	9.969	0.602	9.372	0.573	10.449	0.225
RetinexNet [36]	6.705	0.324	6.714	0.450	7.009	0.311	6.805	0.454	6.584	0.197
RT-X Net [37]	10.235	0.415	10.521	0.448	10.874	0.432	10.236	0.529	11.145	0.375
IAT	15.163	0.679	15.485	0.633	15.487	0.705	15.407	0.686	16.021	0.669

**Table 2:** Experimental Environment.

Hardware/Software Used	Description
CPU	Intel Core Ultra 9-275HX
GPU	NVIDIA GeForce RTX 5080
GPU Parallel Processing Platform	CUDA 12.8
CUDA Deep Neural Network Library	cuDNN v 8.9.7
Operating System	Windows 11
Programming Language	Python 3.12.7
Deep Learning Framework	PyTorch 2.7.1
Integrated Development Environment	PyCharm 2025.1.3.1-Windows

**Table 3:** Experimental Results under Different Models.

Model	Precision (P)	Recall (R)	mAP@0.5	mAP@0.5:0.95	GFLOPs	Memory (G)	Inference Times (ms)
Faster R-CNN	0.852	0.876	0.915	0.574	181.4	36.2	29.56
YOLOv8	0.874	0.881	0.923	0.582	28.80	11.7	11.41
YOLOv11	0.880	0.884	0.935	0.594	21.70	7.39	9.85
YOLOv12	0.903	0.910	0.938	0.603	19.60	7.02	6.73
YOLO-ILM	0.948	0.941	0.957	0.689	45.2	9.92	9.43

**Table 4:** Ablation Test Results.

Test	IAT	LEGM	MPDIoU	Precision (P)	Recall (R)	mAP@0.5	mAP@0.5:0.95	GFLOPs	Inference Times (ms)
1				0.903	0.910	0.938	0.603	19.60	6.73
2	√			0.909	0.912	0.942	0.632	24.20	6.88
3		√		0.916	0.914	0.944	0.649	27.30	8.87
4			√	0.914	0.915	0.940	0.641	19.60	7.13
5	√	√		0.929	0.925	0.958	0.657	45.20	9.31
6	√		√	0.930	0.934	0.952	0.663	24.20	8.92
7		√	√	0.936	0.929	0.949	0.676	27.30	9.34
8	√	√	√	0.948	0.941	0.957	0.689	45.20	9.43

**Table 5:** Improvement in Each Module of the Ablation Experiments.

Module Combination	ΔPrecision(%)	ΔRecall (%)	ΔmAP@0.5 (%)	ΔmAP@0.5:0.95 (%)
Baseline	–	–	–	–
IAT	0.60	0.20	0.40	2.90
LEGM	1.30	0.40	0.60	4.60
IAT + LEGM + MPDIoU	1.10	0.50	0.20	3.80
IAT + LEGM	2.60	1.50	2.00	5.40
IAT + MPDIoU	2.70	2.40	1.40	6.00
LEGM + MPDIoU	3.30	1.90	1.10	7.30
IAT + LEGM + MPDIoU	4.50	3.10	1.90	8.60

## Data Availability

The original contributions presented in this study are included in this article; further inquiries can be directed to the corresponding author.

## References

[1] Moreau K., Laamanen C., Bose R., Shang H., Scott J.A. (2021). Environmental impact improvements due to introducing automation into underground copper mines. Int. J. Min. Sci. Technol..

[2] Tang J., Yang H., Chen H., Li F., Wang C., Liu Q., Zhang Q., Zhang R., Yu L. (2024). Potential and future direction for copper resource exploration in China. Green Smart Min. Eng..

[3] Wang Y., Chen Q., Dai B., Wang D. (2024). Guidance and review: Advancing mining technology for enhanced production and supply of strategic minerals in China. Green Smart Min. Eng..

[4] Liang G., Liang Y., Niu D., Shaheen M. (2024). Balancing sustainability and innovation: The role of artificial intelligence in shaping mining practices for sustainable mining development. Resour. Policy.

[5] Shiryayeva O., Suleimenov B., Kulakova Y. (2025). Sustainable Mineral Processing Technologies Using Hybrid Intelligent Algorithms. Technologies.

[6] Quan Z., Sun J. (2025). A Feature-Enhanced Small Object Detection Algorithm Based on Attention Mechanism. Sensors.

[7] Korie J., Gabriela C.-A., Ezeonyema C., Oshim F. (2024). Machine Learning Innovations for Improving Mineral Recovery and Processing: A Comprehensive Review. Int. J. Econ. Environ. Geol..

[8] Corrigan C.C., Ikonnikova S.A. (2024). A review of the use of AI in the mining industry: Insights and ethical considerations for multi-objective optimization. Extr. Ind. Soc..

[9] Tao M., Lv S., Feng S. (2023). Study on the Evaluation of the Development Efficiency of Smart Mine Construction and the Influencing Factors Based on the US-SBM Model. Sustainability.

[10] Shen X., Li L., Ma Y., Xu S., Liu J., Yang Z., Shi Y. (2025). VLCIM: A vision-language cyclic interaction model for industrial defect detection. IEEE Trans. Instrum. Meas..

[11] Liu Y., Wang X., Zhang Z., Deng F. (2023). LOSN: Lightweight ore sorting networks for edge device environment. Eng. Appl. Artif. Intell..

[12] Shatwell D.G., Murray V., Barton A. (2023). Real-time ore sorting using color and texture analysis. Int. J. Min. Sci. Technol..

[13] Tsangaratos P., Ilia I., Spanoudakis N., Karageorgiou G., Perraki M. (2025). Machine learning approaches for real-time mineral classification and educational applications. Appl. Sci..

[14] Hui Y., Wang J., Li B. (2024). WSA-YOLO: Weak-supervised and Adaptive object detection in the low-light environment for YOLOV7. IEEE Trans. Instrum. Meas..

[15] Zhou Y., Zhang T., Li Z., Qiu J. (2025). Improved Space Object Detection Based on YOLO11. Aerospace.

[16] Zhang Q., Guo W., Lin M. (2025). LLD-YOLO: A multi-module network for robust vehicle detection in low-light conditions. Signal Image Video Process..

[17] Zhang M., Yue K., Li B., Guo J., Li Y., Gao X. (2024). Single-Frame Infrared Small Target Detection via Gaussian Curvature Inspired Network. IEEE Trans. Geosci. Remote Sens..

[18] Wang S., Jiang H., Li Z., Yang J., Ma X., Chen J., Tang X. (2024). PHSI-RTDETR: A Lightweight Infrared Small Target Detection Algorithm Based on UAV Aerial Photography. Drones.

[19] Wang Z., Su Y., Kang F., Wang L., Lin Y., Wu Q., Li H., Cai Z. (2025). PC-YOLO11s: A Lightweight and Effective Feature Extraction Method for Small Target Image Detection. Sensors.

[20] Fang W., Chen W. (2025). TBF-YOLOv8n: A Lightweight Tea Bud Detection Model Based on YOLOv8n Improvements. Sensors.

[21] Peng D., Ding W., Zhen T. (2024). A novel low light object detection method based on the YOLOv5 fusion feature enhancement. Sci. Rep..

[22] Das P.P., Ganguly T., Chaudhuri R., Deb S. (2025). YOLO-D: A Domain Adaptive approach towards low light object detection. Procedia Comput. Sci..

[23] Liu Q., Huang W., Hu T., Duan X., Yu J., Huang J., Wei J. (2025). Efficient network architecture for target detection in challenging low-light environments. Eng. Appl. Artif. Intell..

[24] Han Z., Yue Z., Liu L. (2025). 3L-YOLO: A Lightweight Low-Light Object Detection Algorithm. Appl. Sci..

[25] Ye S., Huang W., Liu W., Chen L., Wang X., Zhong X. (2025). YES: You should Examine Suspect cues for low-light object detection. Comput. Vis. Image Underst..

[26] Qi Z., Wang J., Yang G., Wang Y. (2025). Lightweight YOLOv8-Based Model for Weed Detection in Dryland Spring Wheat Fields. Sustainability.

[27] Zhao Y., Li W., Yang R., Liu Y. (2025). Real-time efficient image enhancement in low-light condition with novel supervised deep learning pipeline. Digit. Signal Process..

[28] Qiu Z., Huang X., Sun Z., Li S., Wang J. (2025). GS-YOLO-Seg: A Lightweight Instance Segmentation Method for Low-Grade Graphite Ore Sorting Based on Improved YOLO11-Seg. Sustainability.

[29] Li J., Meng X., Wang S., Lu Z., Yu H., Qu Z., Wang J. (2025). Enhanced Parallel Convolution Architecture YOLO Photovoltaic Panel Detection Model for Remote Sensing Images. Sustainability.

[30] Tian Y., Ye Q., Doermann D. (2025). Yolov12: Attention-centric real-time object detectors. arXiv.

[31] Cui Z., Li K., Gu L., Su S., Gao P., Jiang Z., Qiao Y., Harada T. (2022). You only need 90k parameters to adapt light: A light weight transformer for image enhancement and exposure correction. arXiv.

[32] Zhang Y., Zhou S., Li H. Depth information assisted collaborative mutual promotion network for single image dehazing. Proceedings of the IEEE/CVF Conference on Computer Vision and Pattern Recognition.

[33] Ma S., Xu Y. (2023). Mpdiou: A loss for efficient and accurate bounding box regression. arXiv.

[34] Li Y., Fu C., Liu T., Hu Z., Song G. (2025). Two-stage remaining useful life prediction method across operating conditions based on small samples and novel health indicators. Reliab. Eng. Syst. Saf..

[35] Guo C., Li C., Guo J., Loy C.C., Hou J., Kwong S., Cong R. Zero-reference deep curve estimation for low-light image enhancement. Proceedings of the IEEE/CVF Conference on Computer Vision and Pattern Recognition.

[36] Wei C., Wang W., Yang W., Liu J. (2018). Deep retinex decomposition for low-light enhancement. arXiv.

[37] Jha R., Lenka A., Ramanagopal M., Sankaranarayanan A., Mitra K. (2025). RT-X Net: RGB-Thermal cross attention network for Low-Light Image Enhancement. arXiv.

[38] Zheng Z., Wang P., Ren D., Liu W., Ye R., Hu Q., Zuo W. (2022). Enhancing Geometric Factors in Model Learning and Inference for Object Detection and Instance Segmentation. IEEE Trans. Cybern..

[39] Ren S., He K., Girshick R., Sun J. (2017). Faster R-CNN: Towards Real-Time Object Detection with Region Proposal Networks. IEEE Trans. Pattern Anal. Mach. Intell..

[40] Varghese R., Sambath M. Yolov8: A novel object detection algorithm with enhanced performance and robustness. Proceedings of the 2024 International Conference on Advances in Data Engineering and Intelligent Computing Systems (ADICS).

[41] Khanam R., Hussain M. (2024). Yolov11: An overview of the key architectural enhancements. arXiv.

